# Some Facts Concerning Human Hybridity

**Published:** 1881-03

**Authors:** Harvey L. Byrd

**Affiliations:** 225 North Gilmor Street; Baltimore, Md.


### Independent Practitioner.

VOL. II.——*March, 1881.*——No. 3.

ARTICLE I.

### SOME FACTS CONCERNING HUMAN HYBRIDITY.

BY HARVEY L. BYRD, A. M., M. D., etc., etc., Baltimore, Md.

Much importance is attached, by some of those who hold the doctrine of unity of origin of the human races, to certain expressions of the Apostle Paul, and particularly so to what our version of the New Testament makes him say to the Athenians on Mars' Hill, viz:— That "God hath made of one blood all nations of men to dwell on all the face of the earth." But when the original is examined, and the translation carefully compared with the facts, as they exist, it will be seen that the learned apostle intended to convey in unmistakable language the idea of a plurality of races, but all created by one God. The Greeks believed themselves the autochthones of their country, and consequently admitted no relationship whatever, with the Hebrew or Jewish race, to which Paul belonged.

No one was more familiar with their belief than the apostle was himself; and the sentence as it stands in the Greek certainly does not favor the idea of an original unity of races; as the word αἱματος which has been translated "blood" in the quotation above, is said to be wanting in more than a dozen of the ancient and principal manuscripts. The text reads in the Latin Vulgate, as follows, viz: "*Fecit que ex uno omne genus hominum inhabitare super universam faciem terræ.*" A proper translation of this sentence will prove that the apostle merely intended to show that every race of men, had its origin in God, and was descended from Him, who made them all to dwell upon the earth. The translation is in these words: "And hath made of one every race of men to dwell on all the face of the earth." Paul knew that this was the right argument to use to a people who believed they originated, or sprang, from the soil on which they had lived from immemorial time. So much for the inference the unitists attempt to draw from the argument before the Athenians. The great and radical differences and distinctions which have so markably characterised the Caucasian, and Negro races, for the past five thousand years, and maintain their integrity now, without any perceptible, or appreciable change, is prima facie evidence, at least, that they have existed the same from the period of their original creation. If then the varying conditions of climate and circumstances, through this long period of centuries, have effected absolutely no change in their physical condition, including the amount and shape of cerebral matter, it is but natural that we should neither look for, nor expect, any in their mental capacity and ability, or equality in mental activity.

The Negro in his native land and circumstances, has never written an intelligible line, nor carved and erected a monument to his fellow man! On the other hand, the evidences of Caucasian intelligence, and moral grandeur of the white man, are around him everywhere upon the earth's surface which he has selected for his abode! We see clearly in all this, the impress and control of law; and are therefore not compelled to grope very far into the shadowy past, for a solution of the matter under consideration. The Caucasian has ever been the highest type, and the Negro the lowest of the genus-homo. The inter-breeding of those two distinct races, like the breeding in a single race, has developed laws which govern their progeny with unerring certainty. Were the two races descended from a common stock, such laws would not be called into operation, and nothing would, as a consequence, interfere with their breeding, *inter se*, indefinitely, and the production of a strong and prolific progeny. But is such inter-breeding attended with these results?

Some of the unitists lay great stress upon this point, and contend there is neither natural law nor physical hindrance against such inter-breeding; and introduce this assertion in support of their theory of the common origin of the races, that the progeny resulting from inter-breeding of the white and colored races, is a prolific one; and thus attempt to disprove the Hybridity of the mulatto. It would be an useless expenditure of time, and the space of the Journal, to follow the objectors to the plural origin of the races, through all their ramifications, and having given prominence to their most salient points, we will proceed as briefly as practicable to a statement of a few facts in support of our argument on the diversity of origin of the races ot men. The fecundity of the mulatto is known to all familiar with that Hybrid, but the constant tendency to physical *degeneracy* of its offspring seems not to have attracted the attention to which its importance entitles it. Partaking, and blending together, the physical, and other characteristics of the white, and negro races, to a great extent; but inferior in physical strength, and endurance to either of the parent races, the mulatto is endowed with larger cranial capacity, and consequently, greater mental scope, and power than its negro progenitor. He inherits, with his increase of mental endowment over the negro, a constitutional tendency to scrofula, and other hereditary predispositions to the diseases of his progenitors, thus rendering him liable to a greater number than either of them; but he is more protected, against certain epidemic diseases than is either race of his ancestors, e. g.:—

He is more exposed to cholera, as it has prevailed on the rice-plantations of the South, than the white race, and less so than the negro; and more liable to yellow fever than the negro, and much less so than the caucasian! Thus, *cæteris paribus*, the measure of his white blood is the measure of his liability to yellow fever, and that of his negro blood, his protection against cholera during epidemics of those diseases.

The tendency to abortion is very marked, and greatly stronger in the mulatto female than in either white or black woman, under the same, or very similar circumstances; and should she breed in the same degree of blood admixture, or with a mulatto man, the abortive tendency is much increased; and a quadroon female, ($\frac{3}{4}$ white) breeding with a quadroon male, will rarely pass to the full term of utero-gestation.

The difficulty of carrying a conception in the octoroon female, ($\frac{7}{8}$ white) is such, when impregnated by either an octoroon, or quadroon, or even a mulatto male, that it would be safe to say, that not more than one successful parturition, of a viable fœtus, could be calculated upon in a dozen pregnancies, and such a product would be short lived and usually feeble from birth! Whilst endowed with stronger and more insatiable sexual propensity than the negro female, conception is a less frequent result in the mulatto than in her. It is less frequent in the quadroon, with equally strong, if not increased sexualism, than in the mulatto; and is of rarer occurence in the octoroon, than either, when breeding with a man of similar admixture of blood, or with a white man, even. It will thus be seen, that the constant tendency of the human hybrid, is to sterility, degeneracy and extinction. We know of no descendant of octoroon parents twelve years of age, and think it extremely doubtful whether one exists five years old. We have no hesitation in saying that a prolific and permanent hybrid mulatto race, is a physical and physiological *impossibility!*

Such are the facts now, and there is no reason for supposing they have ever been otherwise. To deny these laws would be to rob Deity of half of His attributes and perfections. And if law *ab initio*, does it not establish the truth of our position that no new and permanent race has ever been evolved from the Negro race? Much less one of a markedly *superior* type, as the Caucasian?

If Negro blood when admixed with the Caucasian, as in the Mulatto, cannot, under existing law, impart sufficient strength and physical endurance, to this higher type or variety for its separate existence; with its increase of mental power and capacity; is it probable, or even possible, to suppose that it could in the eons of the past, have given birth to a superior race, as much superior, mentally, to the Mulatto as he is to the Negro race? With the law and facts before us, the answer is emphatically No! The admission of any such hypothesis, would be a quasi endorsement of the totally unproved and mythical theory of animal evolution! But let us see what is said by others, of the product of inter-breeding of the white and black races?

Mr. Edward Norriss says: "All recorded evidence declares Mulattoes, half-casts, to be more liable to disease and of shorter life than either parent, and shows that their inter-marriages are dicidedly less prolific than those of other persons." Col. Charles H. Smith, declares: "We doubt exceedingly if a Mulatto family does, or could exist, in any part of the tropics, continued to a fourth generation from one stock." Dr. Knox says: "With the cessation of the supply of European blood, the Mulatto of all shades must cease." Dr. J. C. Nott, states: "They (Mulattoes) are less prolific than the parent stock; which coupled with an inherent tendency to run out, so much so that mulatto humanity seldom, if ever reaches, through subsequent crossings with white men that grade of dilution which washes out the negro stain." Other authorities might be quoted of like character, but want of space will not now allow of its being done. A word or two, on the diseases and general treatment of the mulatto will close this too brief article. Mulatto males are more predisposed to scrofula and tuberculosis, and succumb more quickly to inflammatory affections than either of the parent stocks: and mulatto females inheriting the same predispositions, and tendencies, as the males, are more prone to certain derangements and diseases of the uterine organs; and hysteria enters largely as a factor in many more cases, than in either the white or black female. Diseases assume an asthenic type at an earlier stage, in the mulatto than in either of the parent races, and as those receive different treatment foı the same diseases, even under the same circumstances, the treatment of the mulatto will, therefore, necessarily vary widely from that which is called for in the races from which he sprang; and might be said to consist of a compromise between what is used in the white and negro races. We are reluctantly compelled to close these necessarily concise remarks on this highly interesting subject, for the present.

225 North Gilmor Street.

